# Russell Body Gastroenteritis: An Aberrant Manifestation of Chronic Inflammation in Gastrointestinal Mucosa

**DOI:** 10.1155/2013/797264

**Published:** 2013-10-03

**Authors:** Feriyl Bhaijee, Keith A. Brown, Billy W. Long, Alexandra S. Brown

**Affiliations:** ^1^Department of Pathology, University of Mississippi Medical Center, 2500 North State Street, Jackson, MS 39216, USA; ^2^GI Associates & Endoscopy Center, 1405 North State Street Suite 308, Jackson, MS 39202, USA

## Abstract

First described in 1998, Russell body gastritis is a rare chronic inflammatory condition characterized by abundant intramucosal polyclonal plasma cells, which contain intracytoplasmic eosinophilic globules of immunoglobulins (Russell bodies) that displace the nucleus, with an accompanying chronic inflammatory infiltrate. Russell bodies represent a cellular response to overstimulation of plasma cells, leading to the accumulation of abundant, nondegradable, condensed immunoglobulin in dilated rough endoplasmic reticulum cisternae. Russell body gastritis usually occurs in the gastric antrum, but two cases of Russell body duodenitis have been recently described. Herein, we report an unusual case of Barrett esophagus with prominent lymphoplasmacytic infiltration and Russell bodies, which expands the current spectrum of Russell body gastritis/duodenitis. Given the various anatomic locations in which Russell body gastritis may arise, we suggest that “Russell body gastroenteritis” may be a more appropriate designation for this uncommon reactive condition.

## 1. Introduction

First described in 1998 [1], Russell body gastritis (RBG) is a rare inflammatory condition characterized by abundant intramucosal polyclonal plasma cells, which contain intracytoplasmic, eosinophilic globules of immunoglobulins (Russell bodies) that displace the nucleus, with an accompanying chronic inflammatory infiltrate. It usually occurs in the gastric antrum, but two cases of Russell body duodenitis have been recently described. Herein, we report an unusual case of Barrett esophagus with prominent lymphoplasmacytic infiltration and Russell bodies, which expands the current spectrum of Russell body gastritis/duodenitis.

## 2. Case Report

**Figure 1:**
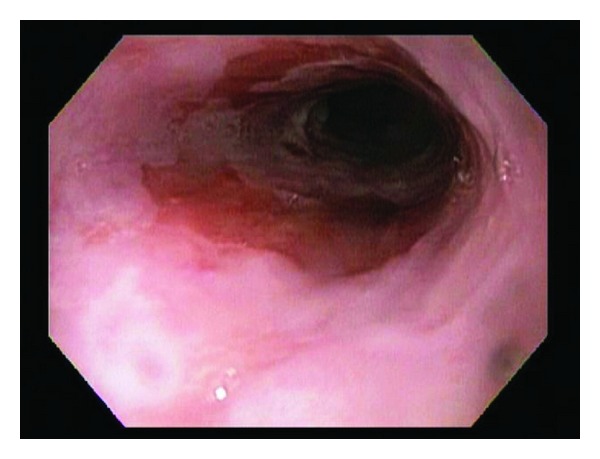
Upper endoscopy (esophagogastroduodenoscopy): a residual 1–1.5 cm band of salmon-pink Barrett mucosa separated from the gastroesophageal junction by endoscopically unremarkable squamous mucosa.

**Figure 2:**
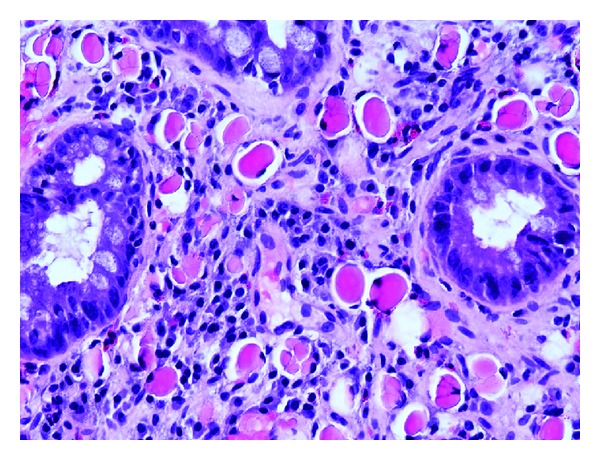
Hematoxylin and eosin, 20x. Biopsies from the Barrett mucosa showed intestinal metaplasia with active inflammation and numerous monomorphic cells in the lamina propria with eccentric nuclei and abundant eosinophilic cytoplasm.

**Figure 3:**
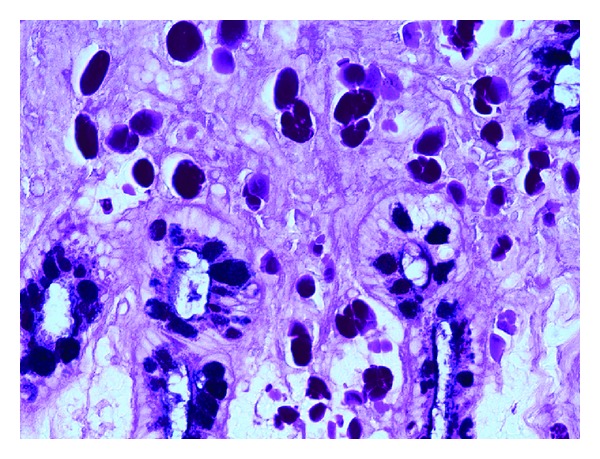
Periodic acid-Schiff-Alcian blue stain (pH 2.5), 20x. The monomorphic cells were highlighted by a periodic acid-Schiff (PAS) stain; note the dark blue inhomogeneous staining of intracytoplasmic mucin in the intestinal-type metaplastic goblet cells, which is characteristic in Barrett mucosa.

**Figure 4:**
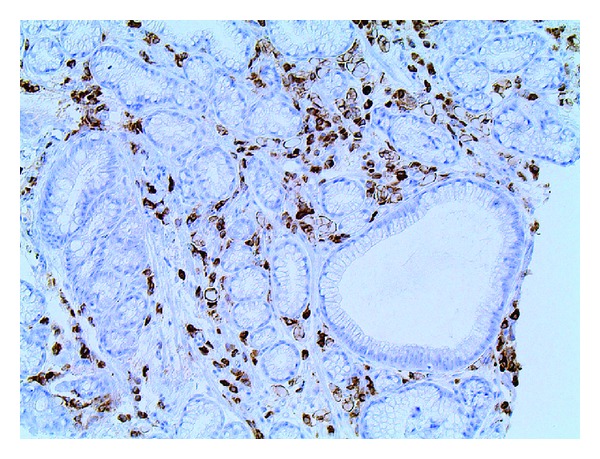
CD79a immunostain, 20x. The distended protein-containing cells in the lamina propria are highlighted by CD79a, a plasma cell marker.

**Figure 5:**
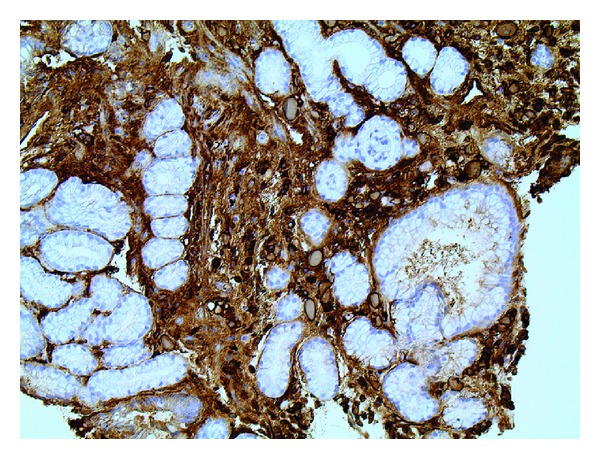
Kappa light chain immunostain, 20x. The Russell body-laden plasma cells show both kappa and lambda (i.e., polyclonal) immunoglobulin expression.

**Figure 6:**
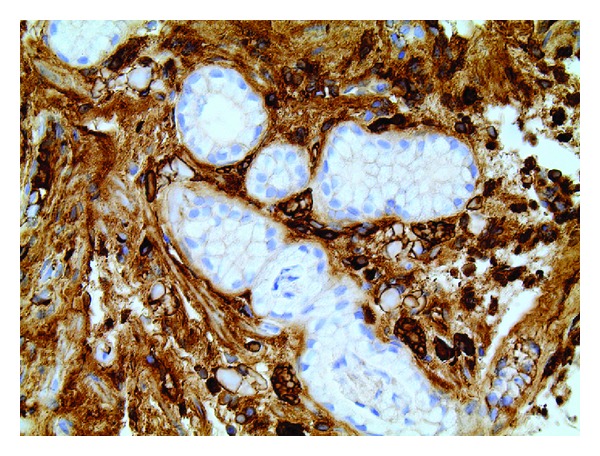
Lambda light chain immunostain, 40x. The Russell body-laden plasma cells show both kappa and lambda (i.e., polyclonal) immunoglobulin expression.

A 69-year-old male with a history of Barrett esophagus (6 cm length of involvement) underwent an ablation procedure, which resulted in a residual 1–1.5 cm band of Barrett mucosa (Figure 1) that was separated from the gastroesophageal junction by endoscopically unremarkable squamous mucosa. Two years later, biopsies from the residual band of Barrett mucosa showed intestinal metaplasia with active inflammation and numerous monomorphic cells in the lamina propria with eccentric nuclei and abundant eosinophilic cytoplasm (Figure 2). The monomorphic cells were highlighted by a periodic acid-Schiff (PAS) stain and immunohistochemical studies for CD79a, kappa, and lambda (Figures 3, 4, 5, and 6). There was no immunoreactivity with pan-cytokeratins (AE1/AE3). This immunohistochemical profile supported that these cells were polyclonal plasma cells, as seen in Russell body gastritis (RBG). The Barrett mucosa was called indefinite for dysplasia due to active inflammation. 

## 3. Discussion

Russell body gastritis/duodenitis is an unusual form of chronic gastrointestinal mucosal inflammation, characterized by abundant plasma cells containing eosinophilic cytoplasmic globules. Russell bodies represent a cellular response to overstimulation of plasma cells, leading to the accumulation of abundant, nondegradable, condensed immunoglobulin in dilated rough endoplasmic reticulum cisternae [2]. Plasma cells filled with abundant intracytoplasmic Russell bodies are called Mott cells, which can be seen in disease states characterized by plasmacytosis and chronic inflammation, such as chronic follicular gastritis, autoimmune-mediated diseases such as Hashimoto's thyroiditis and rheumatoid arthritis, and hematopoietic tumors with plasmacytic differentiation, such as MALT lymphoma, plasmacytoma, or lymphoplasmacytic lymphoma [3]. Mott cells are extremely rare in epithelial tumors. In gastrointestinal mucosal plasmacytic infiltrates, the absence of nuclear atypia, mitotic activity, lymphoepithelial lesions, and monoclonal infiltrates, favors a benign, reactive process, such as chronic inflammation.

Including our current case, there are 24 reported cases of Russell body gastritis, duodenitis, and Barrett esophagitis in the English medical literature (Table 1) [1, 3–22]. The mean age of affected patients in these reports is 61 years (range 34–88 years), with a male-to-female ratio of 2.4 : 1. Most patients presented with nonspecific gastrointestinal symptoms, such as abdominal discomfort, nausea, and dyspepsia. Endoscopic features were likewise non-specific, including mucosal erythema, edema, erosion, ulceration, or, rarely, raised nodules. Biopsy specimens from all cases showed active chronic inflammation with either focal or diffuse accumulation of plasma cells containing Russell bodies. Of the 20 gastric cases, 12 showed evidence of *Helicobacter pylori* infection; all 4 extragastric (duodenal and esophageal) cases were *H. pylori* negative. Other associated conditions included HIV infection [9, 11, 16, 20], ethanol abuse [1, 5], gastric carcinoma [14, 19], Barrett esophagus [22], monoclonal gammopathy of uncertain significance [8], and concurrent Hepatitis C infection and insulin-dependent diabetes mellitus [17].

While plasmacytic infiltrates are the hallmark of gastric chronic inflammation, plasma cells containing Russell bodies are rare in *Helicobacter pylori*-associated gastritis. It is hypothesized that chronic *Helicobacter* infection stimulates plasma cell-driven hyperproduction of immunoglobulins, which leads to Russell body formation and Mott cell proliferation. The highly pathogenic *Helicobacter pylori *genotypes vacA and cagA may also be associated with RBG [23]. The disappearance of Russell bodies following successful *Helicobacter pylori* eradication therapy also supports the etiopathogenic role of the organism in RBG [1, 3, 7, 10, 11, 13, 15]. In 2006, Stewart and Spagnola reported three cases of *Helicobacter*-associated gastritis with crystalline plasma cell inclusions, which may represent another morphologic manifestation of immunoglobulin accumulation in response to chronic gastritis [24].

While RGB is usually a benign incidental finding associated with *Helicobacter* infection [1, 6, 11], it has also been described in association with gastric tubular [19] and signet ring cell [14] adenocarcinoma. Russell bodies are actually more frequently seen in normal tissues adjacent to malignant processes compared to benign conditions, which suggests a possible association between malignancy and Russell body formation [25]. Coexistence of gastric carcinoma and RBG is not entirely unexpected, given the frequent association between RBG and chronic *Helicobacter* infection—an established risk factor for gastric cancer.

In 2010, Shinozaki et al. [26] reported two cases of lymphoepithelioma-type, EBV-associated gastric carcinoma with extensive lymphoplasmacytic infiltration and prominent Mott cells [26]. EBER in situ hybridization highlighted the tumor cells obscured by the Mott cell proliferations, and the authors postulated that aberrant chemokine expression in the EBV-driven tumors led to plasma cell activation and subsequent Russell body formation and Mott cell proliferation. Although both cases also showed evidence of *H. pylori *infection, the Mott cell proliferations were confined to the mucosa involved by the tumors and were not seen in the background mucosa; thus, RBG was excluded.

Russell body duodenitis has recently been reported in two patients: a 55-year-old HIV-infected male [20] and a 69-year-old female [21] with autoimmune disease. In both cases, the Russell body infiltrates and chronic inflammation occurred in areas of duodenal gastric metaplasia and, notably, in the absence of demonstrable *Helicobacter* infection. In this setting, Russell body duodenitis may represent either the residual sequela of healed or partiallytreated gastric *Helicobacter* infection or an abnormal response to disordered systemic immune responses. In 2005, Rubio described Mott cells in Barrett esophagus [22]. To our knowledge, the current case is the second reported case of RBG in Barrett esophagus. Regardless of anatomic location, it is likely that chronic inflammation causes aberrant chemokine expression, resulting in overstimulation of plasma cells, excessive immunoglobulin production, and subsequent Russell body formation.

RBG represents a potential diagnostic pitfall because the distended plasma cells may be mistaken for signet ring tumor cells [4]. The plasma cells in RBG, however, lack nuclear atypia, mucicarmine, and cytokeratin expression. The periodic acid-Schiff reaction may help identify Russell bodies by conferring a dense, glassy stain to intracytoplasmic immunoglobulins. Plasma cell markers, such as CD138 and CD79a, and concomitant kappa and lambda light chain expression will demonstrate the polyclonal nature of the plasma cell infiltrate. Associated gastric carcinoma and infectious agents, such as *Helicobacter* and *Candida*, may alter patient management and clinical outcome and therefore should be excluded by ancillary studies.

The differential diagnosis for plasma cell infiltrates in the lamina propria of the luminal gastrointestinal tract including plasma cell neoplasms, such as plasmacytoma and mucosa-associated lymphoid tissue (MALT) lymphoma with plasmacytic differentiation. Benign Mott cell proliferations can be distinguished from hematopoietic malignancies by the absence of cellular atypia, mitotic activity, and monoclonality. Immunohistochemistry, in situ hybridization, and PCR for immunoglobulin heavy chain rearrangements aid in the evaluation of clonality.

The management of RBG involves *Helicobacter* eradication therapy and exclusion of associated conditions, including other infectious agents and gastric carcinoma.

In conclusion, we report the second case of Barrett esophagus with prominent lymphoplasmacytic infiltration and Russell bodies, which expands the current spectrum of Russell body gastritis/duodenitis. Given the various anatomic locations in which Russell body gastritis may arise, we suggest that “Russell body gastroenteritis” may be a more appropriate designation for this uncommon reactive condition.

## Figures and Tables

**Table 1:** Reported cases of Russell body gastritis, duodenitis, and Barrett esophagus.

Case	Authors	Age/sex	Location	*H. pylori* infection	Other conditions	Country
1	Yu et al. 1987 [4]	65/F	Stomach	Unknown	Unknown	Korea
2	Tazawa and Tsutsumi 1998 [1]	53/M	Stomach	Yes	Ethanol abuse	Japan
3	Erbersdobler et al. 2004 [5]	83/F	Stomach	No	Esophageal candidiasis, ethanol abuse	Germany
4	Ensari et al. 2005 [6]	70/M	Stomach	Yes	—	Turkey
5	Paik et al. 2006 [7]	47/F	Stomach	Yes	—	Korea
6	Paik et al. 2006 [7]	53/F	Stomach	Yes	—	Korea
7	Wolkersdörfer et al. 2006 [8]	54/M	Stomach	Yes	MGUS	Germany
8	Drut and Olenchuk 2006 [9]	34/M	Stomach	No	HIV+	Argentina
9	Pizzolitto et al. 2007 [10]	60/F	Stomach	Yes	—	Italy
10	Eum et al. 2007 [3]	48/M	Stomach	Yes	Colonic polyps	Korea
11	Licci et al. 2009 [11]	59/M	Stomach	Yes	HIV+	Italy
12	Habib et al. 2010 [12]	75/M	Stomach	No	Renal failure, dyslipidemia, ethanol abuse, and prior rhabdomyolysis	USA
13	Del Gobbo et al. 2011 [13]	78/F	Stomach	No	—	Italy
14	Wolf et al. 2011 [14]	67/M	Stomach	Yes	Signet ring cell carcinoma	Austria
15	Yoon et al. 2012 [15]	57/M	Stomach	Yes	Gastric & colonic polyps	Korea
16	Yoon et al. 2012 [15]	43/M	Stomach	Yes	—	Korea
17	Bhalla et al. 2012 [16]	82/M	Stomach	No	HIV+	USA
18	Coyne and Azadeh 2012 [17]	49/M	Stomach	No	Hepatitis C+, IDDM	UK
19	Karabagli and Gokturk 2012 [18]	60/M	Stomach	Yes	—	Turkey
20	Choi et al. 2012 [19]	55/M	Stomach	Yes	Gastric adenocarcinoma	Korea
21	Savage et al. 2011 [20]	55/M	Duodenum	No	HIV+, lymphoma	USA
22	Mondolfi et al. 2012 [21]	69/F	Duodenum	No	Crohn's disease, cirrhosis, rheumatoid arthritis, and obesity	USA
23	Rubio 2005 [22]	88/M	Esophagus	No	Barrett esophagus	Sweden
24	Bhaijee et al.	71/M	Esophagus	No	Barrett esophagus	USA
